# Follow-Up Investigation of 41 Children After Metallic Airway Stent Implantation: An 8-Year Experience

**DOI:** 10.3389/fped.2020.579209

**Published:** 2020-10-26

**Authors:** Meng Wang, Bin Zhu, Xuan Xu

**Affiliations:** Bayi Children's Hospital, Affiliated to the 7th Medical Center of Chinese PLA General Hospital, Beijing, China

**Keywords:** metal stent, tracheal stenosis, children, complication, follow-up

## Abstract

**Objective:** To present 8-year follow-up outcomes, treatment of complications, and prognosis in children with congenital tracheal stenosis after metallic airway stent implantation.

**Methods:** Retrospective analysis was performed on the clinical records of children who had airway stents placed between May 20, 2011 and May 31, 2016, and on their follow-up records collected on November 31, 2019.

**Results:** During the 8 years follow-up, 41 children underwent airway stenting under flexible bronchoscopy and participated in the follow-up investigation. There were 26 cases with left main bronchus (LMB) stenosis (63.4%), 16 cases with congenital tracheal stenosis (CTS, 39.0%), 12 cases with right main bronchus (RMB) stenosis (29.3%), and 1 case of subglottic stenosis (2.4%). A total of 76 stents were implanted, and 21 patients died after implantation. There were 34 children (82.9%) with congenital heart disease (CHD), while other diseases accounted for <5%. Among children with CHD and those with other conditions, the number of death cases was 19 and 2, respectively; no significant differences were observed between the two groups (P>0.05). The most frequent complications were increased airway secretion (75.8%), stent deformation (66.7%), and granulation tissue hyperplasia (60.6%). The airway stenosis (45.5%) and stent migration (12.1%) occurred at a moderate rate. The less common complications were airway softening (6.1%), and stent breakage (6.1%).

**Conclusions:** The placement and removal of the metallic stent are convenient and quick procedures that can relieve the symptoms of dyspnea caused by airway stenosis in the case of an emergency. After stent implantation, the primary disease should be actively treated, and the stent should be removed as soon as possible after the cause of airway stenosis is successfully removed. Larger stents are more likely to cause complications; thus, a good follow-up system should be established to timely address all the complications.

## Introduction

Congenital tracheal stenosis and tracheomalacia are congenital tracheal stenosis that causes airway obstruction (1) and are commonly encountered in pediatric intensive care units (PICU), especially in infants (2). In these children, pleural pressure exceeds intraluminal pressure resulting in airway collapse during dynamic expiration and coughing at low lung volume (3). They often have severe symptoms and require mechanical ventilation, while clinical management is difficult and challenging (4). Patients with severe airway obstructions require corrective treatments that guarantee sufficient airstream. Currently, there are several surgical treatments and non-surgical interventions available for this condition. Surgical procedures have a relatively high re-operational rate for stenosis and mortality from later airway complications. An attractive non-surgical option is endoscopic airway stenting (5). These stents can provide rapid relief of dyspnea in children, and tend to improve clinical symptoms within a few hours after the patient is taken off the ventilator. Yet, airway stents can irritate the trachea wall, causing varying degrees of complications. For example, hyperplasia of granulation tissue can lead to recurrence and even death caused by tracheal stenosis (6). The goal of this study is to report the 8-year follow-up outcomes, treatment of complications, and prognosis in children with congenital tracheal stenosis after metallic airway stent implantation.

## Materials and Methods

A retrospective analysis was performed on the clinical records of children who had airway stents placed at Bayi Children's Hospital (Beijing, China) between May 20, 2011 and May 31, 2016, and on their follow-up records collected by November 31, 2019. Informed consent was obtained from the child's guardian for all cases.

A total of 41 children were diagnosed with severe central airway stenosis by fiberoptic bronchoscopy combined with CT and three-dimensional airway reconstruction, and all underwent airway stenting under soft bronchoscopy. All stents were placed through a 2.8 mm bronchoscope (Olympus BF-XP260F or BF-XP60, Olympus Optical Co Ltd, Tokyo, Japan) with a 1.2 mm working channel, positioned in the airway segment, and expanded. The stents were retrieved through the fiberoptic bronchoscope with pliers (Olympus Optical Company Ltd, FB52C-1, 1050 mm length, 2 mm diameter) under multifunctional ECG monitoring and oxygen administration via a nasal catheter. Based on the granulation tissue hyperplasia, argon plasma coagulation (APC), CO_2_ cryotherapy, balloon dilatation, and other treatment plans were selected to treat granulation tissue (7).

The study protocol was approved by the ethics committee of the 7th medical center of Chinese PLA General Hospital (2020-017).

### Follow-Up

If patients had no difficulties breathing after the operation, they returned for a follow-up visit once a month. Patients were encouraged to contact the Department of Bronchology for examination and treatment if breathing difficulties re-occurred.

### Indications for Stent Placement

When lumen reduction was within 80%, stent placement was recommended in cases with weaning failure, apnoea episodes, frequent respiratory infections, severe respiratory distress, or failure to thrive.

### Indications for Stent Removal

The stents were usually removed 2 to 3 months after implantation; the placement time could be extended according to the condition. Indications for stent removal were: (1) tracheal examination revealing obvious granulation tissue hyperplasia around the stent; (2) stents abstracting discharge airway secretions or led to lung infections; (3) the stent was broken.

### Stent Types

Balloon expandable metallic angioplasty rapamycin coated stents (Partner, Beijing, China) were used for airway stenting. The stents were made of stainless-steel mesh and were readily available in different sizes. The stent length was based on the measured length of the narrowed tracheal or bronchial segment, and the stent diameter was chosen by reference to the diameter of the adjacent normal trachea or bronchus.

### Statistical Analysis

Data were analyzed using SPSS version 22.0 software (IBM Armonk, New York, NY, USA). Continuous variables were reported as mean, standard deviation, minimum, and maximum, as appropriate. A Chi-square test was used for count data. A *P* < 0.05 was considered statistically significant.

## Results

A total of 41 children were included during the study period, including 26 male children with an average age of 186.9 ± 86.7 days, and 15 female children, 240.0 ± 207.3 days (Table 1).

**Table 1:** Basic indexes for children with airway stenosis.

**Sex**	***n***	**%**	**Ages (days)**
Male	26	63.41%	186.9 ± 86.7
Female	15	36.59%	240.0 ± 207.3

According to the imaging and tracheal endoscopic examination, among all children with airway stenosis, LMB was the most important stenosis site in 26 cases, accounting for 63.41%, followed by 16 cases of CTS (39.0%), 12 cases of RMB (29.3%) and 1 case of subglottic stenosis (2.4%) (Table 2).

**Table 2:** The location of airway stenosis in children.

**Airway stenosis position**	***n***	**%**
LMB	26	63.41%
CTS	16	39.02%
RMB	12	29.27%
Subglottic	1	2.44%

*LMB, left main bronchus; CTS, congenital tracheal stenosis; RMB, right main bronchus*.

Among all children, CHD was the most common primary disease, accounting for 82.9% (*n* = 34). There were 4 (9.8%) children with congenital heart disease complicated with airway malformation. Other diseases were less common, and they accounted for <5% (Table 3).

**Table 3:** Type of primary disease.

	***n***	**%**
CHD	34	82.93
Airway dysplasia	11	26.83
BPD	2	4.88
Laryngomalacia	1	2.44
HPS	1	2.44
Cardiopulmonary resuscitation	1	2.44
Airway web	1	2.44
SGS	1	2.44
Burn	1	2.44

*CHD, congenital heart disease; BPD, bronchopulmonary dysplasia; HPS, hemophagocytic syndrome; SGS, subglottic stenosis*.

Among the 34 cases with CHD, there were 20 cases with a left-right shunt, which mainly included VSD, PDA, and PDA. The right-left shunt was identified in 6 cases, and it mainly involved TOF, TGA, pulmonary stenosis, single ventricle, right aortic arch. There was no shunt in 8 cases, which included pulmonary artery sling, double aortic arch, PAH, APVC, right aortic arch, vessel ring (Table 4). In addition, 11 children with CHD were implanted with airway stents before the operation because they could not tolerate anesthesia. It was not possible to wean off the ventilator the remaining 23 children with CHD after surgery, while 10 of them were successfully weaned off after airway stenting.

**Table 4:** Types of congenital heart disease (*n* = 34).

	**CHD**	***n***
Left-right shunt	VSD + Other types	9
	VSD	1
	VSD + PDA	1
	VSD + PDA + Other types	2
	PDA + Other types	3
	PDA	2
	ASD	2
Right-left shunt	TOF	2
	TGA	1
	TGA + VSD + PDA	1
	ASD + Single ventricle	1
	ASD + Pulmonary stenosis + Right aortic arch	1
No shunt	Pulmonary artery sling	4
	Double aortic arch	1
	PAH + APVC	1
	Right aortic arch	1
	Right aortic arch + Vessel ring	1

*CHD, congenital heart disease; VSD, ventricular septal defect; ASD, atrial septal defect; PDA, patent ductus arteriosus; TOF, tetralogy of Fallot; TGA, transposition of the great arteries; APVC, anomalous pulmonary venous connection; PAH, pulmonary arterial hypertension; Other types: ASD, DORV, single ventricle, pulmonary arterial hypertension, double aortic arch, transverse aortic constriction, pulmonary artery sling, double outlet right ventricle*.

In this study, balloons were placed via a fiberoptic bronchoscope through the dilatation of metallic stents. Among 76 stents placed in 41 children, there were 14 cases with LMB in 1.4 per capita, 9 cases of LMB merger placing 2.2 RMB per capita, 11 cases of CTS in 1.9 per capita, 3 cases of CTS with LMB in 2.3 per capita, 2 cases of CTS merger RMB per capita in 2, 1 case of RMB into 1, and 1 case of SGS into 3 (Table 5). After airway stent placement, 19 children were successfully weaned off the ventilation, 1 showed improved clinical symptoms, and 21 showed no improvement. All the children experienced different degrees of complications after stent implantation, which mainly included secretion increase, stent deformation, granulation tissue hyperplasia, airway stenosis, pneumonia, airway stenosis, airway softening, stent breakage, and airway stenosis. All 21 children who died had a poor primary therapeutic effect, and no deaths were caused by airway stent implantation. Among them, there were nine cases with simultaneous LMB and RMB stenosis, and they had the highest fatality rates (66.7%) (Table 5). During the follow-up period, the most frequent complications were increased airway secretion (75.8%), stent deformation (66.7%), and granulation tissue hyperplasia (60.6%) (Table 6). Airway stenosis (45.5%) and stent migration (12.1%) occurred at a moderate rate. The less common complications were airway softening (6.0%), stent breakage (3.0%), and airway stenosis (3.0%). Further secretions were treated by bronchoscope lavage and atomization inhalation. Apparent granulation hyperplasia was removed as soon as the child was in stable condition so as to reduce the continuous irritation of the airway mucosa by the stent. Some sites with obvious hyperplasia of granulation tissue still required APC, CO_2_ cryotherapy, and balloon dilatation under tracheoscopy. Mild granulation tissue hyperplasia could be observed clinically without affecting pulmonary ventilation. In the case of deformation, migration, and breakage of the stent, the removal of the stent was preferred. After the stent was removed, a new stent could be replaced at the site of airway stenosis, and the granulation tissue hyperplasia could be treated with balloon dilation, APC, and CO_2_ cryotherapy. There were two children with symptoms of airway softening. However, it did not affect the ventilation function of the lung; thus, no special treatment was given.

**Table 5:** Stenosis details, primary disease, outcomes, complication, and prognosis in 41 children with congenital tracheal stenosis.

**Stenosis position**	**Primary disease**	***n***	**Number of stents**	**Number of stents per capita**	**Outcomes**	**Complication**	**Prognosis**
LMB		14	20	1.4			
	CHD	6	9	1.5	4 Weaned off; 2 not improved	4 Secretion increase; 2 Stent deformation; 2 Granulation tissue hyperplasia; 1 Airway stenosis; 1 pneumonia	4 Survival; 2 Death
	CHD + HPS	1	1	1.0	1 Weaned off	1 Secretion increase; 1 Airway stenosis; 1 pneumonia	1 Survival
	CHD + BPD	1	1	1.0	1 not improved	1 Secretion increase; 1 Granulation tissue hyperplasia	1 Death
	CHD + Airway dysplasia	3	6	2.0	3 not improved	1 Secretion increase; 1 Stent deformation	3 Death
	Airway dysplasia	3	3	1.0	2 Weaned off; 1 not improved	1 Secretion increase; 1 Granulation tissue hyperplasia; 1 Stent deformation	2 Survival; 1 Death
LMB + RMB		9	20	2.2			
	CHD	6	13	2.2	1 Weaned off; 5 not improved	3 Secretion increase; 2 Stent deformation; 1 Granulation tissue hyperplasia; 1 Airway stenosis	1 Survival; 5 Death
	CHD+ Cardiopulmonary resuscitation	1	2	2.0	1 not improved	1 Stent deformation; 1 Airway stenosis	1 Death
	Airway dysplasia	2	5	2.5	2 Weaned off	2 Secretion increase; 2 Stent deformation; 1 Granulation tissue hyperplasia	2 Survival
CTS		11	21	1.9			
	CHD	8	12	1.5	4 Weaned off; 4 not improved	5 Secretion increase; 1 Airway softening; 5 Stent deformation; 2 Granulation tissue hyperplasia; 3 Airway stenosis	4 Survival; 4 Death
	CHD+ Airway web	1	1	1.0	1 not improved	1 Secretion increase; 1 Granulation tissue hyperplasia	1 Death
	Airway dysplasia	2	5	2.5	1 Weaned off; 1 not improved	2 Stent deformation; 1Granulation tissue hyperplasia	1 Survival; 1 Death
CTS + LMB	CHD	3	7	2.3	2 Weaned off; 1 not improved	2 Secretion increase; 1 Airway softening; 2 Stent deformation; 1 Granulation tissue hyperplasia; 1 Stents breakage	2 Survival; 1 Death
CTS + RMB		2	4	2			
	CHD	1	2	2.0	1 not improved;	-	1 Death
	CHD+BPD	1	2	2.0	1 Clinical improvement	1 Stent deformation; 1 Stent breakage	1 Survival
RMB	CHD	1	1	1	1 Weaned off	1 Secretion increase; 1 Stent deformation; 1 Airway stenosis; 1 Granulation tissue hyperplasia	1 Survival
SGS	CHD	1	3	3	1 Weaned off	1 Secretion increase; 1 Stent deformation; 1 Granulation tissue hyperplasia	1 Survival

*LMB, left main bronchus; CTS, congenital tracheal stenosis; RMB, right main bronchus; CHD, congenital heart disease; BPD, bronchopulmonary dysplasia; HPS, hemophagocytic syndrome; SGS, subglottic stenosis; Weaned off, weaned off the ventilator*.

**Table 6:** Complications and treatment.

	***n***	**%**	**Days**	**Earliest (days)**	**Latest (days)**	**Resolution**
Secretion increase	25	75.8%	81.32 ± 124.7	1	550	25 Lung lavage; 9 Antimicrobial therapy
Stent deformation	22	66.7%	120.0 ± 212.9	0	884	14 Airway stent removal; 4 No special treatment; 4 CO_2_ cryotherapy; 2 APC
Granulation tissue hyperplasia	20	60.6%	290.8 ± 314.5	28	1168	14 CO_2_ cryotherapy; 9 APC; 9 Airway stent removal;2 No special treatment; 3 Balloon dilatation; 1 Use biopsy forceps
Airway stenosis	15	45.5%	192.9 ± 225.9	1	700	5 Airway stent removal; 5 No special treatment; 4 Replacement of airway stent; 1 Balloon dilatation; 1 APC; 1 CO_2_ cryotherapy
Stents migration	4	12.1%	24.5 ± 45.0	0	92	4 Airway stent removal; 1 APC; 1 Balloon dilatation
Airway softening	2	6.1%	376.0	202	550	2 No special treatment
Stent breakage	2	6.1%	1204.0	894.0	1514.0	2 Airway stent removal; 1 APC; 1 CO_2_ cryotherapy

*APC: Argon plasma coagulation*.

**Figure 1:**
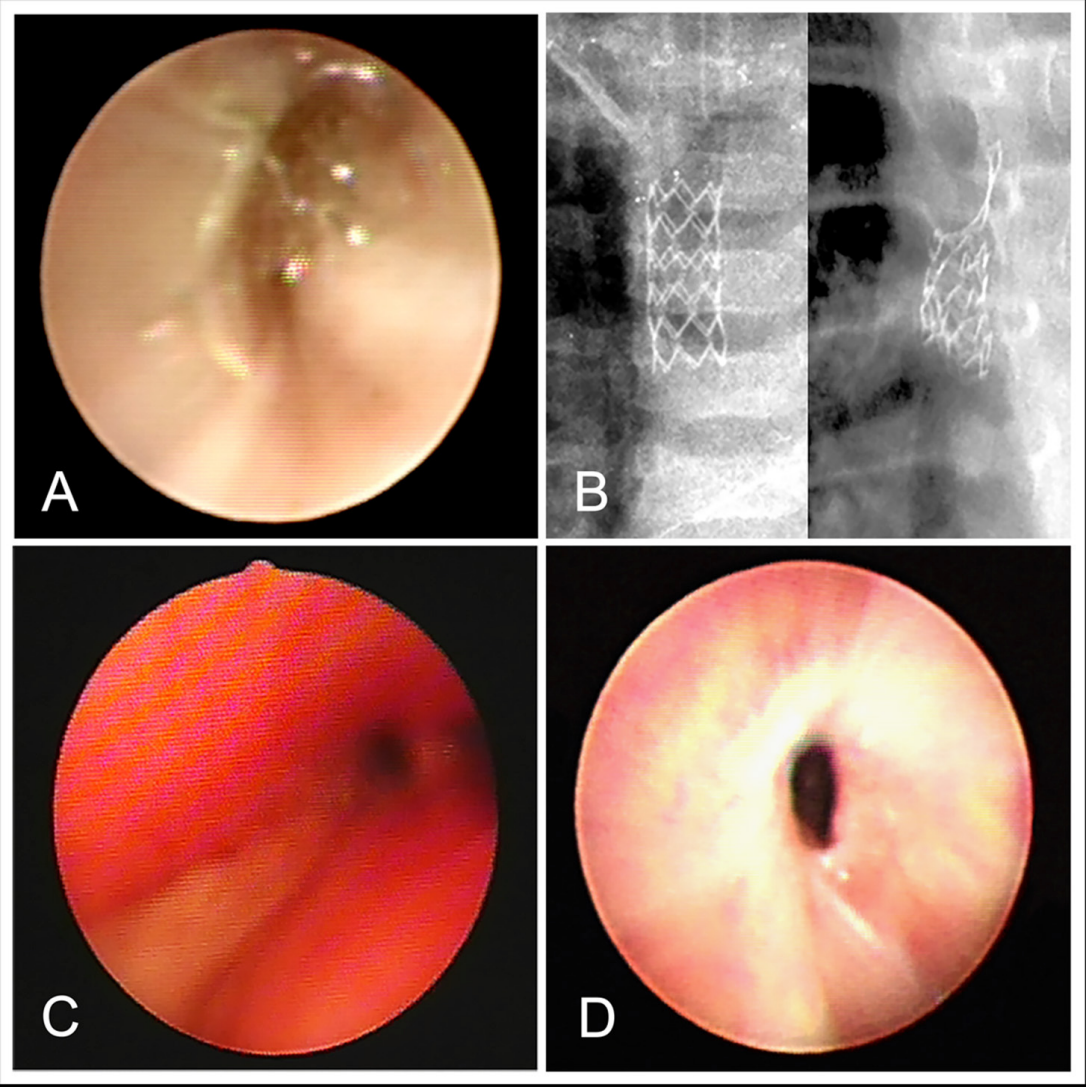
**(A)** Stent deformation. **(B)** Stents breakage. **(C)** Increase of secretion. **(D)** Granulation tissue hyperplasia.

About 50% of the surviving children had stents removed within 1 year, and 8 of them had stents for more than 1 year. The longest time of stent placement was 6 years. As the time of implantation increased, the probability of complications also increased, resulting in the occurrence of complications more than 1 year later in 100% of the children. Complications in the early phase were mainly those caused by stent deformation (Figures 1A,B), migration, and secretion increase (Figure 1C), and a few children developed pneumonia and granulation tissue hyperplasia (Figure 1D). In the mid-term (6–12 months), the main complications were secretion increase, granulation tissue hyperplasia, pneumonia, stent deformation, airway softening, and airway stenosis. The long-term (1–6 years) complications were mainly granulation tissue hyperplasia, airway stenosis, stent deformation, breakage, and airway stenosis (Supplementary Table 1).

If the primary disease of the child could not be timely treated after stent implantation or the cause of airway compression could not be relieved, the death was inevitable (Table 7). Among the 21 children who died after stenting, 13 children died within seven days, 5 children within 7–30 days, and 3 children within 1–8 months. None of the children died from airway stenting (Table 7).

**Table 7:** The cause of death and primary disease in children (*n* = 21).

**Cause of death**	**Primary disease**	***n* (%)**
MOF	5 CHDramya 1 CHD + Airway web	6 (28.6)
Circulatory failure	3 CHD	8 (38.1)
	4 CHD + Airway dysplasia	
	1 CHD + Burn	
Respiratory failure	3 CHDramya 2 Airway dysplasiaramya 1 CHD + BPDramya 1 CHD + Cardiopulmonary resuscitation	7 (33.3)

*MOF, multiple organ failure; CHD, congenital heart disease; BPD, bronchopulmonary dysplasia*.

After stent implantation, the ventilation function of the lung was improved to different degrees. Still, the treatment of the primary disease failed, and the death of the children could not be avoided (Supplementary Table 2).

Among 34 children with CHD and those with other diseases, the number of death cases was 19 and 2, respectively; no significant differences were observed between the two groups (χ^2^ = *0.812, P* > *0.05*). This suggested that CHD was not the main cause of death in children with congenital airway stenosis and that the degree of airway stenosis determined the prognosis in children. Yet, if the primary disease of airway stenosis was not adequately treated, the death could not be prevented.

## Discussion

**Figure 2:**
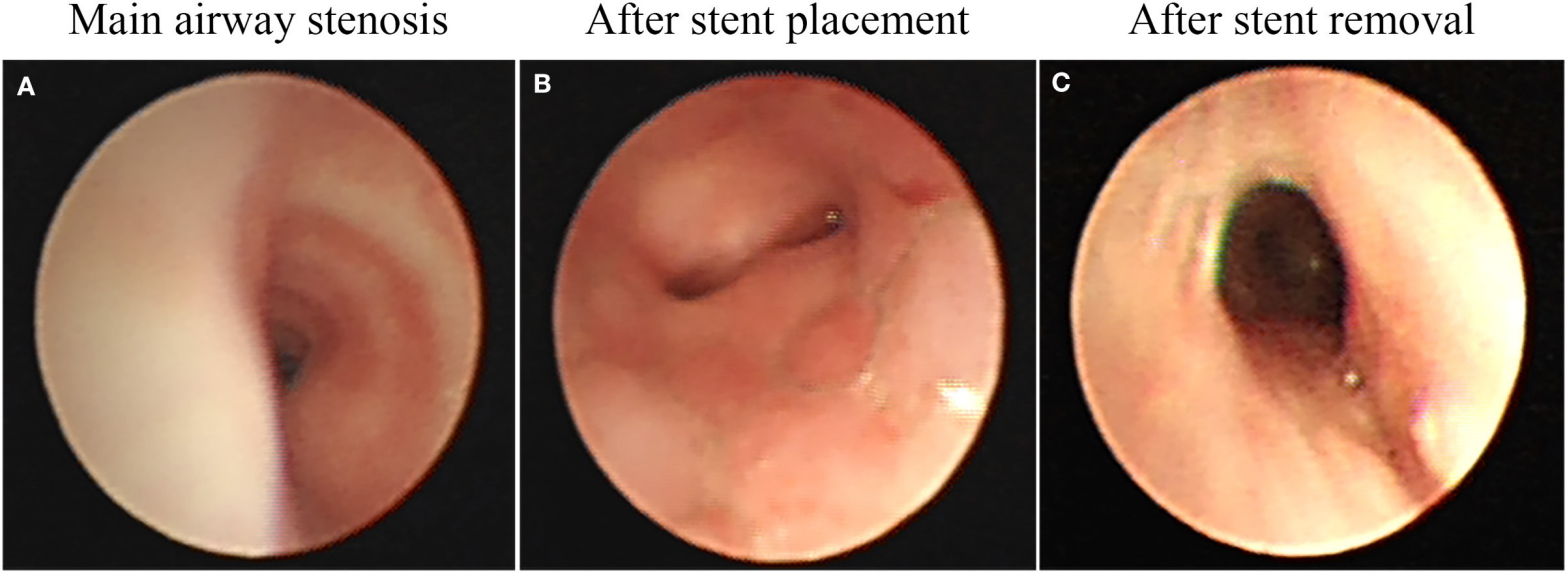
**(A)** Main airway stenosis. **(B)** After stent placement. **(C)** After stent removal.

Our results suggest that the airway stents could be effectively used as a relatively rapid solution for airway stenosis in children (8). Airway stents is a kind of support treatment that can improve symptoms of dyspnea in children, thus creating conditions for treatment of the primary disease (Figures 2A,B). Stents can be removed after effective treatment of the primary disease, after which, lung ventilation is fully restored (Figure 2C). In the present study, most cases with severe airway stenosis were further complicated by CHD (82%), and airway narrowing, which may cause respiratory resistance and energy consumption due to increased breathing. This may delay the heart surgery over a long time, thus increasing the risk of lung infection. Airway stents can relieve airway stenosis; reduce respiration resistance and energy consumption. Yet, they can also lead to ventilator-associated postoperative pulmonary infections. Since children with the airway stenosis and CHD may have affected lung ventilation function, and cannot tolerate anesthesia, they could not undergo preoperative heart surgery via urgent bronchoscopy. Thus, airway stents can be used to create conditions for such operation in these children.

Stent related complications usually occur within the first 2 to 3 months following stent placement, and some, such as stent migration and granulation tissue hyperplasia, may manifest as early as 3 days after the placement (9). In the present study, the most severe complications after stenting (≤ 6 months) were stent migration and deformation, which were observed in nine patients during the first 6 months of follow-up, and in 3 patients after 6 months of follow-up. Moreover, in most cases, the cause of airway compression was not resolved, and the pressure on the airway was much greater than the support provided by the stent. The airway stents can only temporarily solve the problem of airway stenosis. Their purpose is to create the conditions for surgery since their sole use is not enough to solve the airway stenosis. Children with airway obstruction symptoms should immediately undergo bronchoscopy examination, to determine whether a metal stent deformation involved the migration of stents, which in that case should be immediately taken out. A stent may cause airway obstruction after stent implantation, due to the local stimulation, and increased airway secretions, which are relatively common complications. In this study, nine cases (45%) showed increased airway secretions within 1 month, 12 cases (70.6%) had increased airway secretions within 1 month, and 12 cases (70.6%) had increased airway secretions within 1 to 6 months. The clinical symptoms in all these cases could be further improved by the treatment of pulmonary lavage under an atomized tracheoscopy.

**Figure 3:**
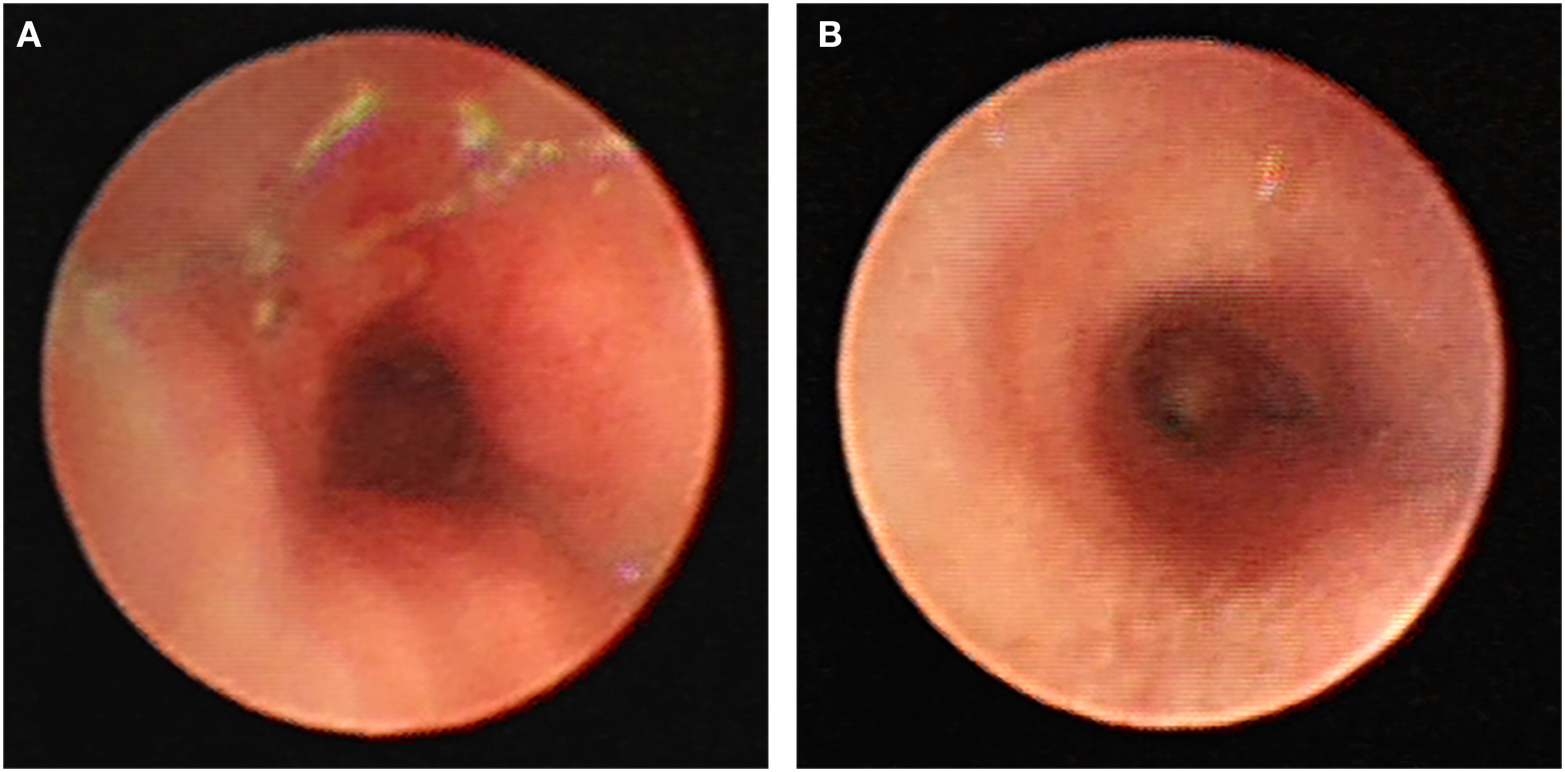
Contrast before **(A)** and 1 week after **(B)** stent removal.

Major long-term complications found in the present study were granulation tissue hyperplasia that occurred in 60% of children after the stent placement. In this study, the earliest granulation tissue hyperplasia appeared on the 28th day after stent implantation. Cases followed for more than 1 year presented with different degrees of complications, with 50% of children having granulation tissue hyperplasia, as the main long-term complication. Mild hyperplasia of granulation tissue generally cannot be treated without affecting ventilation. Patients with granulation tissue hyperplasia caused by airway obstruction or with obvious clinical symptoms should timely undergo a bronchoscope examination. The most recommended treatment is holmium laser, APC, CO_2_ cryotherapy, and balloon dilatation. In the long term, severe granulomatous hyperplasia can make it challenging to remove the stent (Figure 3). When the stent is left for a long time, the granulation tissue tends to wrap most of the stent, thus making it hard to remove the stent using tracheoscopy (10, 11). Chest X-ray, chest CT, airway 3D reconstruction, and other examinations are used for the complete evaluation of the position, shape, and degree of airway stenosis, which then is used to take corresponding treatment measures. It is usually necessary to treat the granulation tissue around the stent through tracheal endoscopic interventional therapy. After the stent is fully exposed, it is clamped with foreign body forceps and removed. Multiple stents can be removed in batches so as to avoid excessive damage to the airway that may occur when multiple stents are removed all at once. After the stent is removed, the wound can be treated with CO_2_ cryotherapy to reduce the rate of granulation tissue hyperplasia (12).

In this study, most of the children had CHD, and most of the airway stenosis and softening in these children were caused by external compression of the trachea or bronchus adjacent to the heart and large vessels caused by the dilation of the pulmonary artery or vascular ring due to the expansion of the heart. Airway stenosis was most common in LMB (63%), followed by CTS (39%). Since these areas are close to the heart and large vessels, they are prone to compression. Children with severe stenosis of both LMB and RMB had a poor prognosis. In this study, there were nine children with severe LMB and RMB stenosis, and only three of them survived.

**Figure 4:**
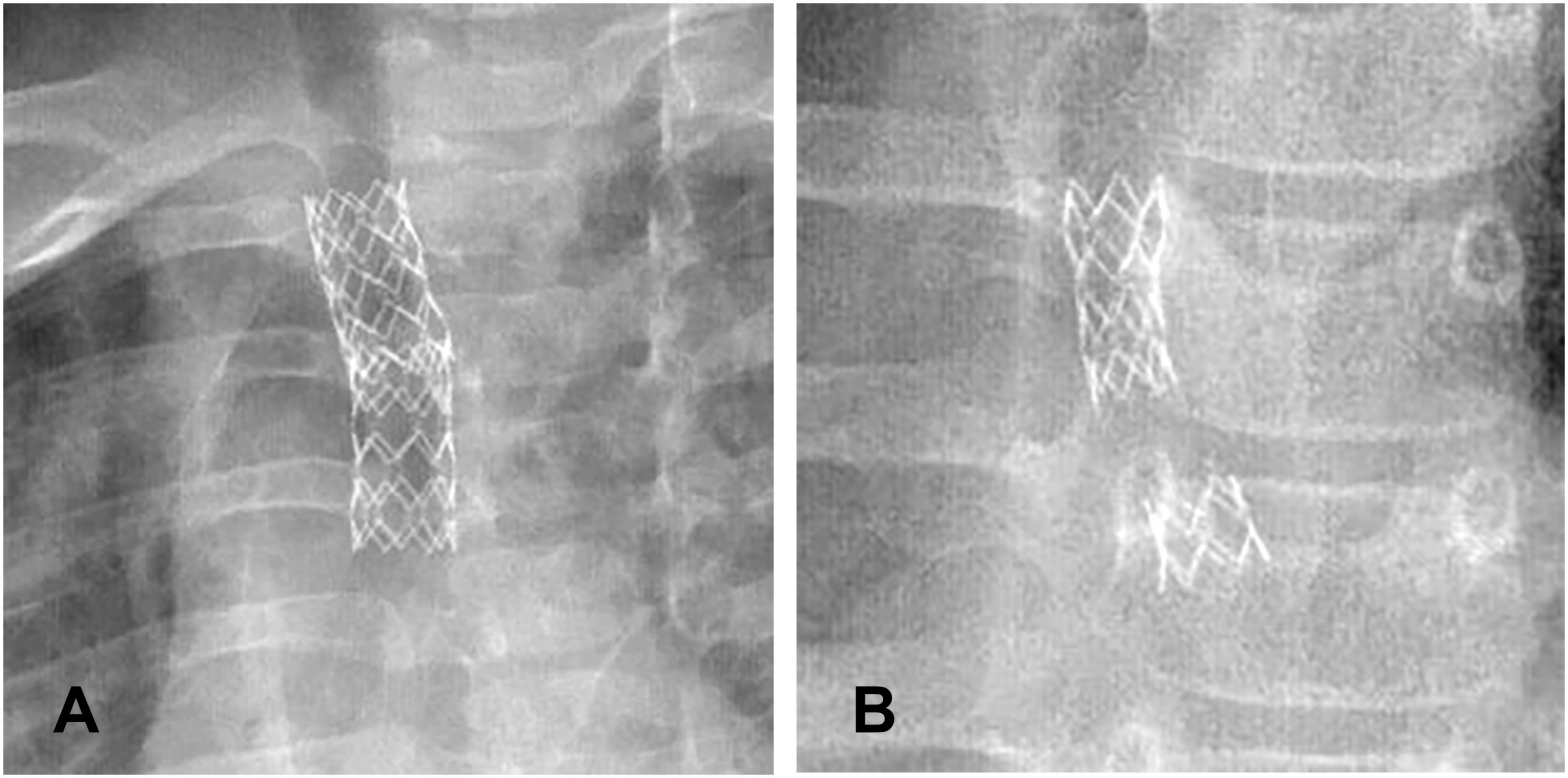
**(A)** Two airway stents overlapping in the central airway. **(B)** Main airway stent breakage.

As a temporary mean of airway support, airway stents should be removed as soon as possible after the etiology of airway compression is relieved. Prolonged stent implantation can lead to hyperplasia of airway granulation tissue, and re-narrowing of the airway, while it also poses a significant risk for stent removal. When the pressure on the airway is greater than the support provided by the stent, the stent tends to deform, move, or even, break. If this should occur, airway problems in children should be re-evaluated, and stents should be replaced with those of more suitable sizes. The number of stents can also be increased to obtain greater support and other measures applied to solve the problem related to airway stenosis (Figure 4A). After the stent is removed, the integrity of the stent should be checked to avoid stent pieces remaining in the airway. In cases with significant granulation tissue hyperplasia around the stent, it is especially tricky to completely remove the stent in one try. When the stent is removed in batches, it tends to break and deform. If the broken stent punctures the airway wall, it may directly cause bleeding of the surrounding large vessels. Sometimes, all the broken stents cannot be found under tracheoscopy, so it is necessary to review the chest X-ray or CT after the stent is removed to ensure that no stent pieces are remaining in the airway. In this study, a patient was implanted with a stent at 3.9 months, and the stent breakage occurred 4.1 years later. During the tracheal endoscopic examination, part of the stent was wrapped by proliferative granulation tissue and pulled (Figure 4B). After the placement of the metal stent, the shape of the stent did not change with the development of the child's airway. When the airway stent is left for a long time, the hyperplasia of granulation tissue may wrap the stent. During the continuous development of the airway, the pulling force of the stent is not uniform, which may result in stent distortion, deformation, and breakage.

Indications for elective stent removal were the following: (1) the time of airway stent placement was 2–3 months. (2) There were no dyspnea and infection, and arterial blood gas analysis suggested no abnormal ventilation and ventilation function. Chest X-rays were normal. Echocardiography showed normal cardiac function. (3) Hyperplasia of granulation tissue was found on bronchoscopy, while there were no signs of dyspnea or pneumonia. (4) The airway stent was deformed and displaced; however, the lung ventilation function was not affected.

Indications for emergency stent removal are: (1) the airway stent was deformed or displaced with dyspnea; (2) breakage of the stent was detected by chest X-ray, chest CT or bronchoscopy.

The advantages of using metal stents include: (1) it can provide better support and can be conveniently and quickly placed under local anesthesia at the ward (13); (2) the mesh of metallic stents helps to preserve mucociliary clearance and, if placed over a bronchial orifice, allows the airstream through its wires. The main limitations of using metal stents are: (1) in long-term stent implantation, granulation tissue hyperplasia may lead to difficult stent removal (14), and airway re-stenosis, which in turn may cause airway bleeding, infection, granulation tissue hyperplasia, subcutaneous emphysema, and other conditions (15); (2) Metallic uncovered expandable stents due to their lack of elastic re-expansion. (3) Airway stent is a foreign body in the airway, which cannot be absorbed by the human body. There are long-term risks related to stent breakage.

The main disadvantages of using nitinol and silicone stents compared to metal stents are: (1) greater mobility compared with metallic stents (16); (2) interruption of mucociliary clearance, mucous plugging, and consequent bacterial growth; (3) prior condition of tracheal inflammation before stent insertion (either for chronic inflammation or for previous surgical or endoscopic treatments on the tracheal mucosa). Nitinol and silicone stents had similar granulation tissue formation rates. Granulation tissue formation has been reported to be more common in metal stents than in silicone stents. Other studies have suggested that granulation tissue is more likely to form in silicone stents.

Previous studies have explored the treatment of biodegradable stents in children with airway stenosis (17). Polydioxanone stents may be an alternative option to metallic or silastic stents in children with collapse or external compression of the trachea (18). Yet, these stents tend to break a few weeks after implanation, causing further damage to the airways. Thus, it has been concluded that compared with metal, nitinol, and silicone stents, the advantages of the biodegradable stent are not obvious.

Furthermore, our analysis of the primary diseases and prognosis in children revealed that there was no higher fatality rate among children with CHD. The severity of airway stenosis, which was not closely related to the primary disease, determined the prognosis in children. In this study, 61.9% of deaths occurred within 7 days after stent implantation, 23.8% occurred within 7–30 days, and 14.3% occurred within 1–8 months.

## Conclusion

Placement and removal of the metal stent are convenient and quick procedures that can relieve the symptoms of dyspnea caused by airway stenosis in the case of an emergency. The primary disease should be actively treated after stent implantation, and the stent should be removed as soon as possible after the cause of airway stenosis is removed. The longer time of stent implantation is associated with more complications; thus, a good follow-up system should be established to timely deal with the complications.

## Data Availability Statement

The raw data supporting the conclusions of this article will be made available by the authors, without undue reservation.

## Ethics Statement

The studies involving human participants were reviewed and approved by the ethics committee of the 7th medical center of Chinese PLA General Hospital (2020-017). Written informed consent to participate in this study was provided by the participants' legal guardian/next of kin. Written informed consent was obtained from the individual(s), and minor(s)' legal guardian/next of kin, for the publication of any potentially identifiable images or data included in this article.

## Author Contributions

XX, MW, and BZ carried out the studies, participated in collecting data, participated in acquisition, analysis, or interpretation of data, and drafting of the manuscript. MW performed the statistical analysis and participated in its design. All authors read and approved the final manuscript.

## Conflict of Interest

The authors declare that the research was conducted in the absence of any commercial or financial relationships that could be construed as a potential conflict of interest.

## Abbreviations

PICU: pediatric intensive care units
APC: argon plasma coagulation.
