# Supplementary material for: Follow-Up Investigation of 41 Children After Metallic Airway Stent Implantation: An 8-Year Experience
Source: Front Pediatr. 2020 Oct 26;8:579209. doi: 10.3389/fped.2020.579209 (PMC7649206; doi:10.3389/fped.2020.579209)
Supplement: Supplementary file 1 [file Table_1.docx]

**Supplementary materials**

| **Table S1.** Complications occurred in the surviving children (n=20) | | | | |
| --- | --- | --- | --- | --- |
| Follow-up period | Number of follow-up | The incidence of complications (%) | | Complications |
| <1 month | 20 | 9 | 45.0% | 6 Secretion increase、2 Stent deformation、2 Stents migration、1 Pneumonia、1 Granulation tissue hyperplasia |
| 1~6 months | 17 | 12 | 70.6% | 7 Secretion increase、6 Granulation tissue hyperplasia、4 Stent deformation、1 Stents migration、1 Pneumonia |
| 7~12 months | 11 | 5 | 45.5% | 2 Secretion increase、2 Granulation tissue hyperplasia、2 Airway stenosis、1 Stent deformation、1 Airway softening |
| 1 ~6 years | 8 | 8 | 100.0% | 4 Granulation tissue hyperplasia、3 Airway stenosis、2 Stent deformation、1 Secretion increase、1 Airway softening、2 Stent breakage |
|  | | | | |
