# Supplementary material for: Follow-Up Investigation of 41 Children After Metallic Airway Stent Implantation: An 8-Year Experience
Source: Front Pediatr. 2020 Oct 26;8:579209. doi: 10.3389/fped.2020.579209 (PMC7649206; doi:10.3389/fped.2020.579209)
Supplement: Supplementary file 2 [file Table_2.docx]

| **Table S2.** The time of death and the condition of primary disease (n=21) | | | |
| --- | --- | --- | --- |
| Time | n | Days | Primary disease |
| < 7 d | 13 | 1.5±1.3 | 8 CHD  2 CHD + Airway dysplasia  2 Airway dysplasia  1 CHD + Airway dysplasia + BPD |
| 7-30 d | 5 | 20.2±6.2 | 3 CHD  1 CHD + Airway dysplasia  1 CHD + Burn |
| 1-8 months | 3 | 140.0±87.5 | 1 CHD  1 CHD + Airway dysplasia  1 CHD + Airway web |
| CHD, congenital heart disease; BPD, bronchopulmonary dysplasia; | | | |
