# Supplementary figures and images for: Follow-Up Investigation of 41 Children After Metallic Airway Stent Implantation: An 8-Year Experience
Source: Front Pediatr. 2020 Oct 26;8:579209. doi: 10.3389/fped.2020.579209 (PMC7649206; doi:10.3389/fped.2020.579209)

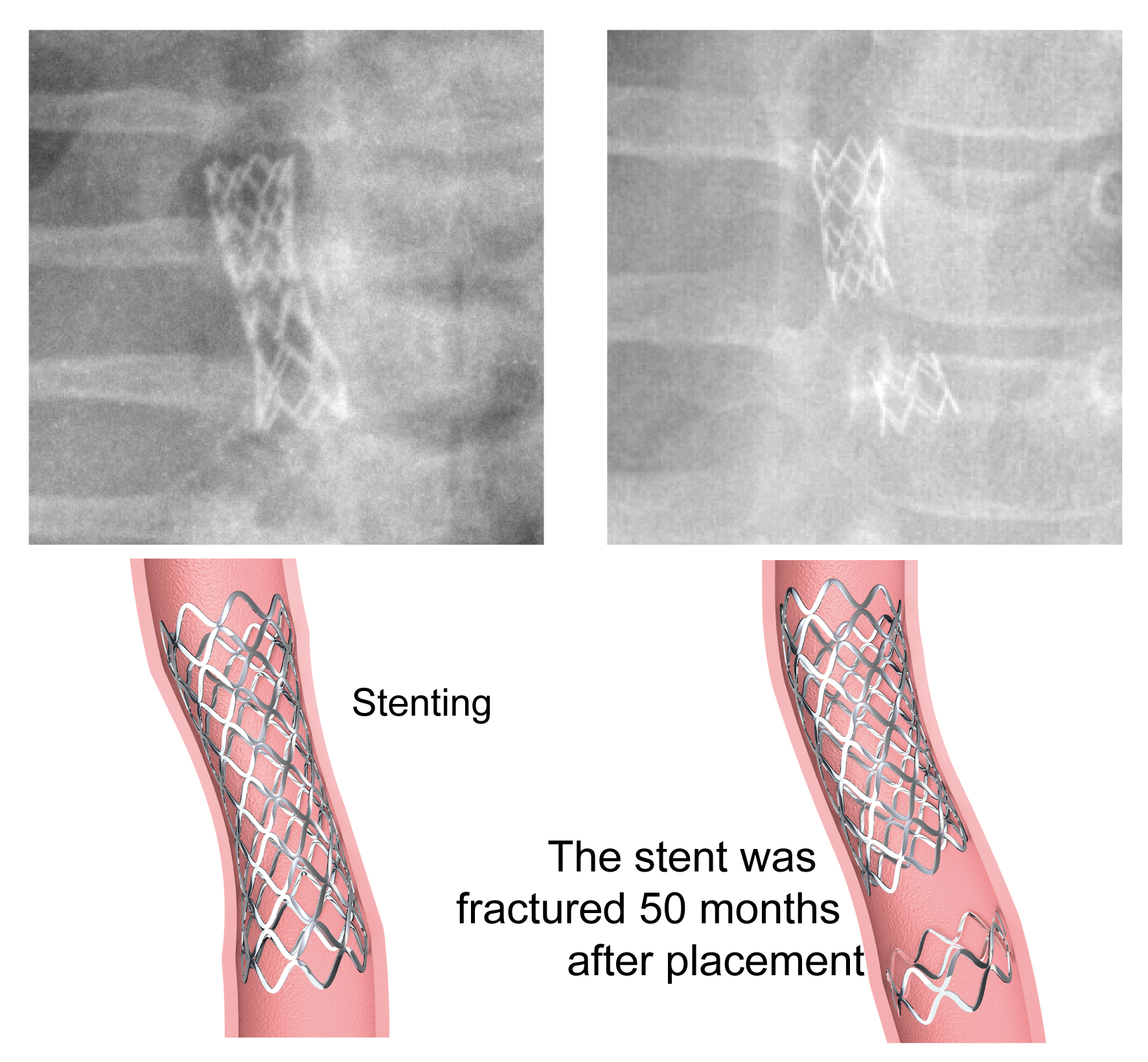

Supplement: Supplementary file 3 [file Image_1.tif]
